# Malicious traffic detection on sampled network flow data with novelty-detection-based models

**DOI:** 10.1038/s41598-023-42618-9

**Published:** 2023-09-18

**Authors:** Adrián Campazas-Vega, Ignacio Samuel Crespo-Martínez, Ángel Manuel Guerrero-Higueras, Claudia Álvarez-Aparicio, Vicente Matellán, Camino Fernández-Llamas

**Affiliations:** 1 Robotics Group, University of León, Campus de Vegazana s/n, 24071 León, Spain; 2 Supercomputación Castilla y León (SCAYLE), Campus de Vegazana s/n, 24071 León, Spain

**Keywords:** Computer science, Information technology

## Abstract

Cyber-attacks are a major problem for users, businesses, and institutions. Classical anomaly detection techniques can detect malicious traffic generated in a cyber-attack by analyzing individual network packets. However, routers that manage large traffic loads can only examine some packets. These devices often use lightweight flow-based protocols to collect network statistics. Analyzing flow data also allows for detecting malicious network traffic. But even gathering flow data has a high computational cost, so routers usually apply a sampling rate to generate flows. This sampling reduces the computational load on routers, but much information is lost. This work aims to demonstrate that malicious traffic can be detected even on flow data collected with a sampling rate of 1 out of 1,000 packets. To do so, we evaluate anomaly-detection-based models using synthetic sampled flow data and actual sampled flow data from RedCAYLE, the Castilla y León regional subnet of the Spanish academic and research network. The results presented show that detection of malicious traffic on sampled flow data is possible using novelty-detection-based models with a high accuracy score and a low false alarm rate.

## Introduction

Cyberattacks on enterprises, organizations, and users have increased in recent years. In addition, the methods used by attackers are also very diverse, making it difficult to detect specific attacks. A promising solution is analyzing network traffic to detect anomalies.

Anomalies are patterns in the data that do not fit a known habitual pattern^1^. We can distinguish between two types of machine learning models to detect anomalies: supervised and unsupervised. Supervised models use previously labeled datasets, while unsupervised models use unlabeled data. There are two types of supervised algorithms for anomaly detection depending on their tolerance. Novelty detection is less tolerant. Novelty detection algorithms expect to receive as input only normal-regular data during training. However, there are more tolerant supervised algorithms. These algorithms can obtain good results even when the dataset contains a small percentage of anomalous data. Outlier detection is the unsupervised technique for anomaly detection. Outlier detection uses regular data with a small ratio of abnormal data to train the models^2, 3^. Models based on anomaly detection have a fundamental advantage over classical supervised learning-based models since their training is based mainly on benign traffic. As a result, these models can detect different network attacks as anomalies with respect to the usual traffic that the model has learned.

Detecting malicious traffic using anomaly models based on machine learning is a problem already solved using network packets. For instance, in^4^, the authors proposed outlier detection algorithms to detect various network attacks. The distance-based outlier detection algorithm obtains an 83.80% accuracy with probing attacks and an 82.21% accuracy with Denial of Service (DoS) attacks. The research in^5^ analyses the use of Local Outlier Factor (LOF) to detect novelties in the NSL-KDD dataset. The authors obtained an 84.00% accuracy score. Finally, in^6^, the authors compared the precision of the Support Vector Machine (SVM) for anomaly detection versus the same model after applying a Principal Component Analysis (PCA) to reduce the dimensionality of the features of the dataset KDD99. The result shows that the SVM applying PCA obtained an accuracy score of 93.75% versus 77.08% obtained without applying PCA.

Studying packets’ payload is an ideal but unrealistic scenario in core routers since studying packets’ payload in real-time is highly CPU-demanding. Therefore, routers that route large volumes of packets cannot analyze all of them in real-time. Usually, routers use flow data instead of packets to analyze network traffic. A flow collects statistics of network-layer datagrams during a specific time interval. All datagrams in a flow have a common set of features, namely source and destination IP address and port^7^. One of the most popular flow-based protocols is NetFlow, developed by Cisco Systems^8^. Flow-based technologies were conceived with the main objective of collecting network statistics and avoiding router saturation. The main objective of their development was not threat detection per se. However, in cases where networks are faced with significant volumes of traffic, and which make an exhaustive analysis of entire network packets unfeasible, these technologies serve as the only recourse to try to identify network-based attacks.

However, some routers manage such a significant volume of network traffic that even collecting flow data is too CPU-intensive. To avoid overloading, these routers process just one out of ${\mathscr {X}}$ packets to gather flow data. This gathering method is known as sampling. While packet sampling alleviates router saturation, also involves a substantial loss of information about network activities^9^.

The number of networks that handle a large amount of traffic and are forced to use packet sampling when generating network flows is increasing. For example, the 5G network, which according to its standard^10, 11^ is expected to manage the traffic of 5.1 billion mobile subscribers and 10 billion IoT subscribers. At a lower order of magnitude, in Spain, RedIRIS – the national network connecting computer resources of universities and research centers – employs NetFlow technology with a sampling rate of 1 packet out of every 1,000 packets to analyze the traffic it handles.

The primary objective of this study is to detect network anomalies, specifically identifying malicious traffic, in networks that utilize network flows and implement sampling of 1 packet out of every 1,000 packets. This specific sampling threshold aligns with the approach utilized in RedCAYLE production routers and has undergone comprehensive analysis in several research papers as can be seen in "Related work" section below. The algorithms employed in this study are founded on the principle of novelty detection. This method has been chosen due to its suitability in scenarios where only a limited quantity of anomalous data is accessible. In networks that use flow data, obtaining accurately labeled malicious flows is a highly intricate task^12^.

### Research motivation

Detecting malicious traffic in networks that use flow-based protocols with packet sampling is a growing requirement in large networks. In particular, our primary motivation is to provide the regional academic network of Castilla y León (RedCAYLE) managers with a solution to detect malicious traffic.

Our proposal aims to identify malicious traffic as network anomalies using flow data with a sampling rate of 1 out of 1,000 packets – The sampling threshold used by Juniper routers that manage RedCAYLE’s traffic –. Additionally, we seek to provide evidence about our results by providing public datasets and tools for replicating or improving them.

This paper also intends to demonstrate that detecting multiple network attacks in sampled network flows using anomaly detection-based techniques is possible. Finally, this work shows that the DOROTHEA tool^13^ can generate NetFlow datasets with high sampling thresholds that can be used to train anomaly detection algorithms for their use on real traffic.

In summary, this paper poses three main contributions:

1. We empirically demonstrate that it is possible to identify malicious traffic as anomalies in network infrastructures collecting flow data with a sampling threshold of 1 out of 1,000 packets. The above has yet to be demonstrated in the literature so far.
2. We experimentally demonstrate that systems trained with datasets generated with DOROTHEA can be successfully used to detect different types of malicious traffic.
3. We provide four datasets with packet-sampled flow data published under a free-use license.

The remainder of the paper is organized as follows: state-the-art of malicious traffic detection with anomaly detection-based methods is posed in "Related work" section; "Materials and methods" section depicts the tools used in our experiments, the data gathering and post-processing, and the optimizing of our detection models as well as the methodology used to evaluate them; results are shown and discussed in "Results and discussion" section; finally, "Conclusions" section pose our conclusions.

## Related work

The identification of malicious traffic as anomalies through the analysis of network packets has been effectively addressed, as previously mentioned. Nevertheless, in networks with high traffic volume, packet analysis becomes impossible, requiring the adoption of flow-based protocols to alleviate the computational burden on routers. Fortunately, in the literature, there have been encouraging outcomes in anomaly detection through the utilization of flow-based datasets.

Ordered from modern to oldest in^14^, the authors propose a hybrid semi-supervised model based on the use of Denoising Auto-Encoder (DAE) and Gate Recurrent Unit (GRU) to detect anomalies in network flows. The results obtained, after evaluating the proposed system with the NSL-KDD dataset, showed an accuracy of 90.21%. In the research carried out in^15^, the authors undertook the task of detecting Distributed Denial of Service (DDoS) attacks in Software Defined Networking (SDN) by leveraging ten distinct features extracted from the CICIDS2017 flow-based dataset. Their approach involved employing anomaly detection techniques, specifically Long Short Term Memory (LSTM) and autoencoder models. The authors achieved an accuracy score of 99.5%. In^16^, the authors demonstrated that the use of the machine learning technique Restricted Boltzmann Machine (RBM) is valid to differentiate between benign and malicious NetFlow traffic when RBM is trained using a balanced dataset. Similarly, the authors in^17^ propose an evolution of the Micro-Clustering Outlier Detection (MCOD) algorithm to detect malicious traffic in NetFlow data. The model used various time series windows and correlations between cluster densities to outline and investigate possible malicious activity in the network, successfully detecting both known and unknown anomalies. The research carried out in^18^ proposes a clustering-based method to detect anomalies in NetFlow traffic. The authors obtained a 96.00% accuracy score in botnet detection. Finally, the research in^19^ presents an approach that leverages SVMs to analyze large volumes of NetFlow records. The results of this work show an average accuracy score in the attack classes studied of around 92.00%.

Previous works used network flows collected without sampling. Networks that handle a very large amount of traffic are forced to sample packets when generating flows. There are some works in the literature that have attempted to detect malicious traffic in network flows collected with different sampling thresholds using machine learning.

In^20^, the researchers investigated the performance of a Decision Tree (DT)-based model designed for detecting malicious traffic using a packet-based and a NetFlow-based dataset. Their study revealed that the adapted DT model achieved comparable accuracy levels when applied to both network packets and flow-based data without any packet sampling. However, a notable decline in accuracy was observed when implementing a sampling rate. With a sampling rate of 1/100, the authors achieved an overall accuracy of 85%. However, when using a more aggressive sampling threshold of 1 packet out of 1,000, the authors found a significant reduction in the capability of the model to detect malicious traffic, resulting in an accuracy score of 50%. Authors in^21^ investigate the influence of packet sampling on the performance of machine learning-based network intrusion systems. They explore three different sampling rates: 1/10, 1/100, and 1/1,000. To conduct their experiments, the authors employ three distinct machine learning algorithms: Convolutional Neural Network (CNN), DT, and Random Forest (RF). The datasets used in their experiments consist of instances of DoS and brute-force attacks. Results show that 50% of the malicious flows are not detected even with a 1/10 sampling rate. In^22^ the authors present a CNN approach for detecting port scans in sampled NetFlow version 5 data. They utilize a graphical representation of flow data to train and evaluate the performance of their system. When using a sampling rate of 1/500, the CNN model achieves an accuracy of 94.15%. However, the authors observe a significant drop in accuracy when employing a more aggressive sampling rate of 1/1,000. Under this condition, the accuracy decreases to 50%

Previous works used approaches based on supervised algorithms. Not many works have been found in the literature using an anomaly detection approach with sampled network flows.

The study carried out in^23^ presents a comprehensive study on whether existing sampling techniques distort traffic features critical for effective anomaly detection. The authors used the sampled data as input to detect two common classes of anomalies: volume anomalies and port scans. The authors used a wavelet-based volume anomaly detection and two hypothesis testing-based port detection algorithms. The experiments were performed with a sampling threshold of 1/10, 1/100, and 1/1,000. The results showed that packet sampling deteriorated the detection capability of the algorithms. At a sampling threshold of 1/1,000, all algorithms lost their detection capability regardless of the sampling technique used.

As mentioned above, the detection of malicious traffic in flow data is possible without packet sampling. However, the situation changes when attempting to apply sampling thresholds similar to those commonly used in production networks, such as RedCAYLE. At the time of writing, there is no existing work that effectively detects malicious traffic as network anomalies when employing such sampling thresholds.

## Materials and methods

This section describes the experiments performed to evaluate our proposal. First, we propose guidelines for gathering flow datasets. Specifically, we depict NetFlow, the protocol used to build flow data. Next, we propose the 2-step data gathering method. On the one hand, we build synthetic flow datasets for fitting our detection models. On the other hand, we collect actual flow data from RedCAYLE to double-check it. Following, we describe the data preprocessing method to prepare the data. Next, we depict the novelty detection algorithms used to build our detection models. Finally, we point out the evaluation method.

### NetFlow

NetFlow^24^ is a lightweight protocol to collect statistical data from network traffic. Cisco Systems released the first version of NetFlow in 1996. NetFlow is popular in gateways that route many network datagrams. In addition, other switches than Cisco’s, such as Juniper’s or Enterasys’, support NetFlow. It provides sufficient information to network administrators to have a high-level understanding of network behavior and possible events occurring on the network. NetFlow supports several versions: V1, V5, and V9. For instance, RedCAYLE uses NetFlow V5. For this version, the collected features are listed in Table 1.

**Table 1:** NetFlow V5 features^25^.

Feature	Description
System uptime	Number of milliseconds since the export device started
Unix-timestamp seconds	Number of seconds since January 1st, 1970 at UTC
Unix-timestamp nanoseconds	Residual nanoseconds since January 1st, 1970 at UTC
Engine type	Flow switching engine type
Engine id.	Slot number switching engine flow
Exporter IP	Flow exporter IP address
Source IP	Source IP address
Destination IP	Destination IP address
Nexthop	Next hop router’s IP address
Input interface	Input interface’s SNMP index
Output interface	Exit interface’s SNMP index
Packets	Number of packets in the flow
Bytes	Sum of bytes of the packets in the flow
First	System uptime of the first packet in the flow
Last	System uptime of the last packet in the flow
Source port	Source port
Destination port	Destination port
Flags	TCP flags
Protocol	IP type of protocol
ToS	IP type of service
Autonomous system source	Autonomous system number of the source, either source or pair
Autonomous system destination	Autonomous system number of the destination, either source or pair
Source mask	Source address prefix mask bits
Destination mask	Destination address prefix mask bits

### DOROTHEA

Docker-based framework for gathering NetFlow data (DOROTHEA) is a tool that uses Docker as a base^13^. DOROTHEA^26^ allows the creation of virtual networks with multiple machines and different structures to gather stream data. DOROTHEA uses a NetFlow sensor to generate streams from network-layer datagrams. The framework consists of two operations. First, the tool allows simulating the generation of benign traffic. Benign traffic generators simulate the network traffic generated by users sending emails, establishing SSH connections, and performing search tasks in web browsers. Then, the traffic goes through the gateway, which performs two main tasks. (1) It routes packets to the Internet, and (2) it sends one out of ${\mathscr {X}}$ packets to the NetFlow Generator. ${\mathscr {X}}$ is the sampling threshold. NetFlow Generator forges NetFlow data from network-layer datagrams. Finally, flows are sent to a NetFlow Warehouse every 2 minutes.

**Figure 1:**
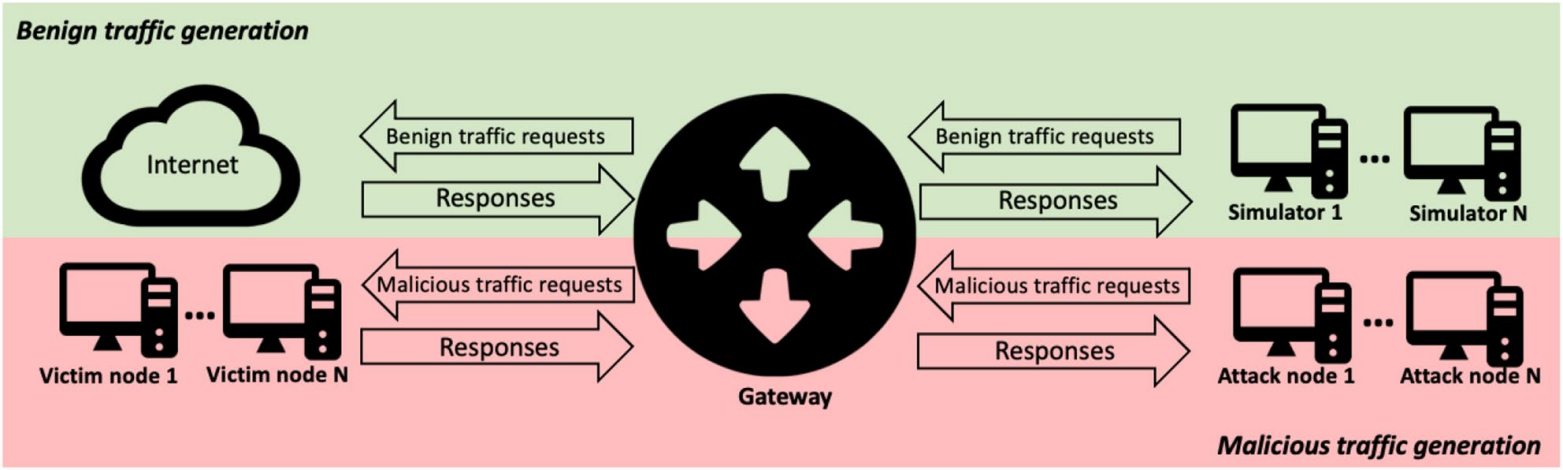
Flow data generation framework.

The second operation allows the simulation of network attacks. It uses an architecture similar to benign traffic generation. This operation is isolated from the Internet, ensuring that all generated flows are malicious. The attacks are carried out in a distributed way using Celery^27^. Celery is a queue-based Python library. The user can define the number of attacker and victim nodes. The attack and benign traffic generator scripts are developed in Python. DOROTHEA allows the user to add new scripts or modify existing ones. Once DOROTHEA has finished, it returns a CSV file containing the generated network flow data. The framework’s architecture is depicted in Fig. 1.

#### Data collection from DOROTHEA

We gathered two datasets using DOROTHEA for fitting and testing novelty detection models. These datasets have been collected using NetFlow with a sampling rate of 1 out of 1,000 packets, simulating the conditions of RedCAYLE’s routers.

The first one (aka ${\mathscr {D}}_1$) only contains benign traffic; this dataset is used to train the models. ${\mathscr {D}}_1$ has been compiled by running three Python scripts available online^28^. The first uses the SMTP protocol to send emails. The second script performs SSH connections as a real user would. Finally, the third script mimics the behavior of a user browsing the Internet. To do this, the script performs queries on various search engines, generating both HTTP and HTTPS traffic. ${\mathscr {D}}_1$ is openly available online^29^.

The second dataset generated (aka ${\mathscr {D}}_2$) contains benign and malicious traffic; this dataset is used to test the models. The malicious traffic corresponds to port scanning attacks and SQL injections. These attacks differ significantly in their structure and nature. SQL injections are classified as application-level attacks, whereas port scanning attacks primarily target the network layer. By incorporating such diverse anomalies, we aim to evaluate the model’s ability to detect a wide range of attacks. Consequently, if the model successfully identifies and classifies these distinct anomalies, it can be inferred that it possesses the capability to detect a multitude of different attacks effectively. Unlike ${\mathscr {D}}_1$, ${\mathscr {D}}_2$ contains approximately 50% benign traffic and 50% malicious traffic. The traffic that simulates a legitimate user has been generated using the same scripts that were used in ${\mathscr {D}}_1$. The generated flows have been labeled as “0”. The malicious flows contained in ${\mathscr {D}}_2$ have been labeled as “1”. The test dataset is also openly available online^30^.

Some of the malicious traffic in the ${\mathscr {D}}_2$ dataset was generated by performing port scanning attacks with the Nmap tool^31^. Specifically, slow port scans have been performed. For this purpose, requests have been launched with a random delay of between 5 and 10 seconds between each request. Attacks were launched on both TCP and UDP protocols. The attacks launched were: TCP SYN scanning; TCP Connect scanning; UDP scanning; TCP NULL; FIN; Xmas scanning; TCP ACK scanning; TCP Window scanning; and TCP Maimon scanning^32^. The attacks were performed from 100 nodes that sent requests to the 65,536 ports of the 200 victim machines.

The remainder of malicious traffic corresponds to SQL injection attacks. To generate these flows, we have deployed web servers in DOROTHEA’s environment that use SQLServer, MySQL, and PostgreSQL as database engines. These servers have been deployed in 200 victim nodes on ports 80, 443, and 8080. The attacks are SQL injection for Union Query and Blind SQL injection. Union Query attack uses Union Operators while inserting the SQL Query. The two SQL queries are joined with the Union Operator. The first statement is a benign query, followed by a malicious query appended with the union operator.

To exploit a Blind SQL injection vulnerability, the attacker launches true or false queries against the database and sets the response based on the response received from the vulnerable application. This attack is performed when the web application is configured to display generic errors without having previously mitigated the SQL injection vulnerability^33^. To generate the flows corresponding to SQL injections, 16 machines have been used to launch attacks against 200 victim nodes.

SQLmap has been used to perform the attacks^34^. The Python scripts are openly available online^35^.

### RedCAYLE

RedCAYLE provides educational centers, university hospitals, scientific infrastructures, and technological facilities with a high-capacity communications backbone network infrastructure, thus allowing access to research network resources and the Internet. In the educational community alone, the network supports more than 380,000 students and teachers from Castilla y León.

RedCAYLE provides several services: 10 Gbps point-to-point transport service, Internet connection, IP addressing, and incident management. Besides, RedCAYLE monitors the affiliated institutions to analyze and diagnose the status of their services. To do so, RedCAYLE uses NetFlow version 5. Using NetFlow allows for a statistics-based analysis since it is impossible to analyze every packet in the network due to computational constraints. However, more than NetFlow is required to avoid overloading RedCAYLE’s routers. Therefore, it is necessary to apply a sampling rate. Specifically, the Juniper MX480 router manufacturer that RedCAYLE uses recommends a sampling threshold of 1 out of 1,000 packets^36^. If this sampling rate is reduced, the manufacturer claims no responsibility for possible breakdowns and problems with the device.

#### Data collection from RedCAYLE

As with the datasets generated with DOROTHEA, two datasets have been gathered from the flows collected in RedCAYLE. The first dataset (aka ${\mathscr {D}}_3$) contains only benign traffic and was used to train the models. However, unlike the traffic collected in DOROTHEA, we cannot claim that the traffic obtained is strictly benign since it does not come from a controlled environment.

A second dataset with malicious and benign traffic has been gathered (aka ${\mathscr {D}}_4$) to test models. To generate the malicious traffic, we carried out new port scanning attacks against nodes within the network range of RedCAYLE. To identify the related flows – and label them as malicious (1) –, the attacks are made from a known IP address range, so all the flows that have an IP address from that range are matched to port scans. Moreover, flows corresponding to benign traffic – label “0” – were selected randomly from the flow data gathered in RedCAYLE. SQL injection attacks have not been included in ${\mathscr {D}}_4$. RedCAYLE is a production network infrastructure and these types of attacks are very intrusive and, therefore, can generate a real risk on a production server.

### Data curation

NetFlow V5 has 24 features. Before training our models, we applied dimensionality reduction. First, we initiate the process by calculating the variance of the features. Variance, as a statistical metric, quantifies the extent of dispersion or variability inherent in a given dataset concerning its arithmetic mean. When a specific feature exhibits a variance of 0, it signifies that the data associated with both malicious and benign traffic for that particular feature are similar and, as a result, do not provide any distinctive information to aid the model’s predictive capabilities. After computing the variance, we removed Exporter IP, Engine type, Engine id, Autonomous system source and destination, and Source and Destination mask features – see Table 1 –. Besides, we removed the Unix-timestamp seconds, and the System uptime of the device, the First and the Last packets in the flow, since the RedCAYLE’s Juniper routers do not send them. In addition, the Unix timestamp seconds has been removed to prevent the models from being affected by the timestamp when the flows were collected. Finally, we removed the Nexthop router’s IP address, according to the conclusion of^37^. In that work, we demonstrated that in a production environment, the Nexthop feature negatively affects the detection of malicious traffic and needs to be removed.

As a result of the above operations, the number of features remaining was 11 – Source IP, Destination IP, Input interface, Output interface, Packets, Bytes, Source port, Destination port, Flags, Protocol, ToS –. To further reduce the dimensionality of the datasets, we applied a PCA. We want to choose the minimum number of dimensions while preserving 95 % of the variance in the dataset. For ${\mathscr {D}}_1$–${\mathscr {D}}_4$, the minimum number of dimensions is 5. As a result, our datasets have five features – computed as a combination of the prior 11 features –.

### Classification model fitting

The models used in the experiment were OC-SVM and iForest. We chose these models because, as seen in "Related work" section, support vector machine-based models and decision tree-based models have demonstrated promising results in malicious traffic detection using both network packets and network flows^6, 19^. Furthermore, these two approaches have also shown promising results when used as supervised algorithms in detecting malicious traffic in sampled flow data^20, 21^. Hence, it is plausible to assume that these models may also yield favorable outcomes in detecting malicious traffic using an anomaly detection-based approach in network flows collected with packet sampling.

OC-SVM is the One-class SVM approach proposed in^38^. The authors proposed adapting the SVM algorithm methodology to the single-class classification problem. After modifying the feature by applying a kernel, they treat the origin as the single member of the second class. The image of a class is separated from the origin using relaxation parameters. Then, standard two-class SVM techniques are employed.

iForest, Isolation Forest is an algorithm inspired by the classification and regression algorithm Random Forest. However, iForest identifies anomalies or outliers. The algorithm isolates observations by selecting a feature and setting the value of the division between the maximum and minimum values of that feature. The division depends on the time it takes to separate the two points. Random partitioning generates significantly shorter trajectories for data that are considered outliers^39^.

The hyperparameters of the models are different when the models are trained on synthetic datasets or on datasets collected from RedCAYLE. This is because anomaly detection models must be fitted with data as close as possible to the data they will find when deployed. Our hyperparameters are shown in Table 2. For the OC-SVM, the value of $\nu$ is both a lower bound for the number of support vector samples and an upper bound for the number of samples on the wrong side of the hyperplane; $\gamma$ specifies the coefficient of the kernel function. For the iForest model, *Contamination* is the amount of pollution in the dataset, and *Trees* specifies the number of base estimators in the ensemble.

**Table 2:** Parameters OC-SVM and iForest models.

Dataset	Classifier	$\nu$	Kernel	$\gamma$	Contamination	Trees
${\mathscr {D}}_1$	OC-SVM	0.042	Polynomial	0.4	None	None
	iForest	None	None	None	0.024	120
${\mathscr {D}}_3$	OC-SVM	0.0029	Polynomial	0.4	None	None
	iForest	None	None	None	0.357	120

We use Model Evaluator (MoEv) to prepare our detection models. MoEv is a general-purpose Scikit-learn^40^ wrapper for building classification models from labeled datasets. MoEv is developed in Python^41^ and provides the following functionalities: data-cleaning, normalization, dimensionality-reduction, and hyperparameter optimization. This optimization is created through GridSearchCV and DASK. DASK provides advanced parallelism, especially useful when using MoEv on a parallel cluster^42^. MoEv trains, evaluates, and gets a report of supervised, semi-supervised, and unsupervised learning-based models. The report includes relevant information such as Accuracy, Precision, Recall, and F1-Score.

MoEv has been used in many different research areas, such as in^43^, where the tool was used to detect jamming attacks in real-time location systems, and in^44^ where the authors predicted academic success in educational institutions. Furthermore, in^13^, MoEV has been validated and used to detect network attacks. To validate the tool, the researchers replicated the work presented in^45^, obtaining similar results.

### Evaluation

To fit and test the iForest and OC-SVM models, we have used the datasets collected with DOTORHEA (${\mathscr {D}}_1$ for training and ${\mathscr {D}}_2$ for testing), and the datasets gathered from RedCAYLE (${\mathscr {D}}_3$ for training and ${\mathscr {D}}_4$ for testing).

To evaluate the experiment, several KPIs were calculated from the confusion matrix generated by each model. First, the accuracy score of the models was calculated as shown in Eq. (1), where $T_P$ is the number of malicious samples correctly identified as malicious. $T_N$ points to the number of harmless or benign samples correctly identified as benign traffic. $F_P$ is the number of benign flows misclassified as malicious. Finally, $F_N$ points to the number of malicious flows misclassified as benign traffic.

1 $$\begin{aligned} Accuracy = \frac{T_P+T_N}{T_P+F_P+T_N+F_N} \end{aligned}$$

Besides the accuracy, we considered the following KPIs obtained through the confusion matrix: False Alarm Rate (FAR), Precision (${\mathscr {P}}$), Recall (${\mathscr {R}}$), and $F_1$-score (${\mathscr {F}}_1$).

FAR is calculated as shown in Eq. (2). FAR is the ratio of false positives and the total number of negative events (regardless of how they were classified).

2 $$\begin{aligned} FAR = \frac{F_P}{T_N+F_P} \end{aligned}$$

${\mathscr {P}}$ computes as shown in Eq. (3). It measures the accuracy of the positive predictions.

3 $$\begin{aligned} {\mathscr {P}} = \frac{T_P}{T_P+F_P} \end{aligned}$$

${\mathscr {R}}$ computes as shown in Eq. (4). It is also called the true positive rate and measures the rate of positive cases correctly identified by the algorithm.

4 $$\begin{aligned} {\mathscr {R}} = \frac{T_P}{T_P+F_N} \end{aligned}$$

${\mathscr {F}}_1$ score computes as shown in Eq. (5). It relates Recall and Precision, being the harmonic mean of both values. While the regular mean treats all values equally, the harmonic mean gives much more weight to low values.

5 $$\begin{aligned} {\mathscr {F}}_1 = 2 \frac{{\mathscr {P}} \times {\mathscr {R}}}{{\mathscr {P}} + {\mathscr {R}}} \end{aligned}$$

## Results and discussion

Firstly, we would like to point out that we have produced a Jupyter notebook available online that allows replicating the experiment performed^46^.

**Table 3:** Dataset volumetry.

Dataset	Source	Aim	Samples	Traffic type
${\mathscr {D}}_{1}$	DOROTHEA	Train	113,195	Benign
${\mathscr {D}}_{2}$	DOROTHEA	Test	15,409	Benign-Malicious
${\mathscr {D}}_{3}$	RedCAYLE	Train	311,936	Benign
${\mathscr {D}}_{4}$	RedCAYLE	Test	921	Benign-Malicious

Table 3 shows the volumetry of the datasets. As shown in the table, the number of flows is higher in the training datasets than in the test sets. This is due to the fact that models based on anomaly detection require a large volume of benign traffic to establish regular patterns. Furthermore, the test datasets are balanced with a malicious traffic percentage of 50% and a benign traffic percentage of 50%. A production network handles far less than 50% of malicious traffic. However, we have used this percentage of traffic to improve the visualization of the results.

The models predict flow by flow, and each prediction is made independently of the others. The balance of the dataset does not exert an influence on the model’s quality. To assert that the balanced dataset exerts no influence, we have conducted experiments employing an imbalanced dataset with a distribution of 99% benign and 1% malicious samples that shows similar results. These tests have not been included in the text for clarity, but can be found in the Jupyter project available. Finally, it is important to note that when a sampling rate of 1 in 1,000 packets is applied, most of the information is lost, and therefore fewer flows are generated.

**Figure 2:**
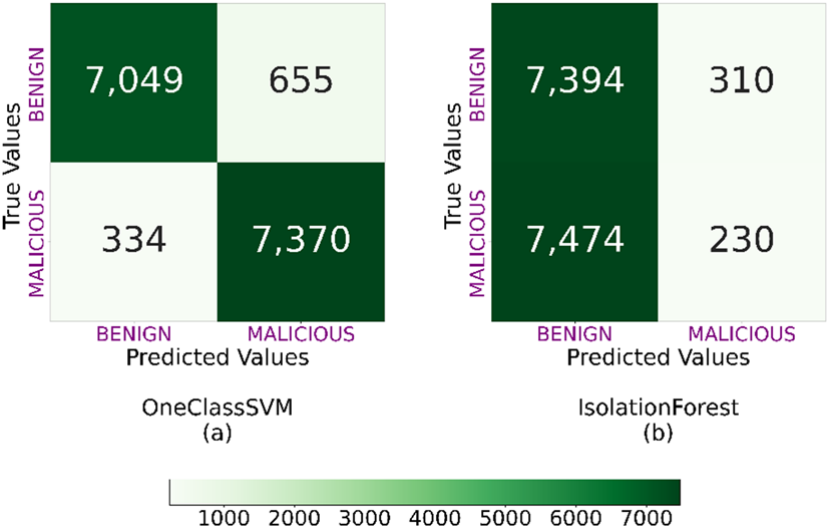
Confusion matrix for OC-SVM (**a**), and iForest (**b**) classifiers tested using ${\mathscr {D}}_2$.

**Figure 3:**
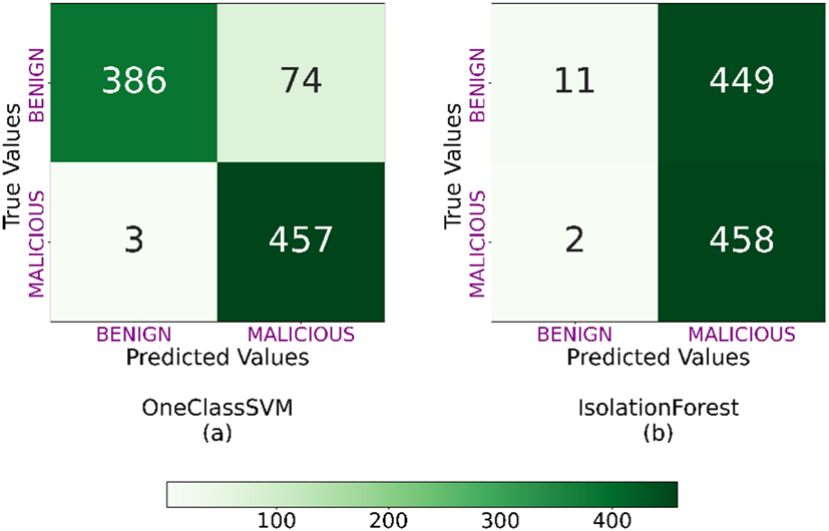
Confusion matrix for OC-SVM (**a**), and iForest (**b**) classifiers tested with ${\mathscr {D}}_4$.

Figures 2 and 3 show the confusion matrices for the OC-SVM and iForest models trained with ${\mathscr {D}}_1$ and ${\mathscr {D}}_3$ and next tested with ${\mathscr {D}}_2$ and ${\mathscr {D}}_4$, respectively. Besides, Tables  4 and 5 show the accuracy, FAR, ${\mathscr {P}}$, ${\mathscr {R}}$, and ${\mathscr {F}}_1$ scores.

**Table 4:** Accuracy, FAR, Precision, Recall and $F_1$-score obtained after testing with ${\mathscr {D}}_2$ the models trained with ${\mathscr {D}}_1$.

Classifier	Accuracy	FAR	Class (1)	${\mathscr {P}}$	${\mathscr {R}}$	${\mathscr {F}}_1$
OC-SVM	0.936	0.082	0	0.955	0.915	0.935
			1	0.918	0.957	0.937
			Avg.	0.937	0.936	0.936
iForest	0.495	0.574	0	0.497	0.959	0.655
			1	0.426	0.029	0.056
			Avg.	0.462	0.495	0.355

(1) Benign flow data is labeled as ‘0’, malicious flow data as ‘1’.

**Table 5:** Accuracy, FAR, Precision, Recall and $F_1$-score obtained after testing with ${\mathscr {D}}_4$ the models trained with ${\mathscr {D}}_3$.

Classifier	Accuracy	FAR	Class (1)	${\mathscr {P}}$	${\mathscr {R}}$	${\mathscr {F}}_1$
OC-SVM	0.916	0.139	0	0.992	0.839	0.909
			1	0.861	0.993	0.992
			Avg.	0.927	0.916	0.916
iForest	0.509	0.495	0	0.846	0.024	0.047
			1	0.505	0.996	0.670
			Avg.	0.676	0.509	0.358

(1) Benign flow data is labeled as ‘0’, malicious flow data as ‘1’.

According to Fig. 2 and Table 4, the best model to detect slow port scanning and SQL injection attacks is the OC-SVM with a 93.6% accuracy score and a low FAR score (0.082). iForest just gets accuracy and FAR scores of 49.5% and 0.574.

Another important KPI in our research is Recall. ${\mathscr {R}}$ shows the rate of positive cases correctly identified by the algorithm, allowing us to know if the algorithm is suitable for detecting malicious traffic, benign traffic, or both. Analyzing ${\mathscr {R}}$, we can observe that the OC-SVM is a balanced model. It detects 95.7% of malicious flow traffic and 91.5% of benign flow traffic. In contrast, ${\mathscr {R}}$ of the iForest models show that these models detect 95.9% of the benign traffic but fail to detect only 3% of the malicious flow traffic.

${\mathscr {F}}_1$ and ${\mathscr {P}}$ follow the same trend as the previous indicators. OC-SVM performs well on both indicators. However, the iForest does not score higher than 50% in either of the two indicators.

Figure 3 and Table 5 show similar results. OC-SVM has the best accuracy score, ${\mathscr {R}}$, ${\mathscr {P}}$ and ${\mathscr {F}}_1$ (higher than 91.6%). In addition, OC-SVM demonstrates a low FAR score (0.139). As with the datasets generated in DOROTHEA, the iForest model is not valid for detecting anomalies in RedCAYLE’s traffic. The model showed an accuracy score of 50.9% and a high FAR score (0.496). ${\mathscr {R}}$, ${\mathscr {P}}$ and ${\mathscr {F}}_1$ do not show good results either.

The above results demonstrated that the OC-SVM model could detect anomalies in networks that gather flow data with a high sampling rate. For example, in our research, 1 out of 1,000 packets. Furthermore, this model has been trained only with benign flow traffic and can detect network attacks that are very different from each other. This fact allows us to speculate that these models could see other network-layer attacks or even 0-day attacks, which means an improvement in the security of this type of network.

Furthermore, looking at Tables  4 and  5, OC-SVM and iForest models obtain similar results both using the synthetic dataset and the dataset collected from RedCAYLE. From this fact, we can state that models based on anomaly detection that provide good results with synthetic datasets generated in DOROTHEA will also provide good results with data coming from production network infrastructures. It is important to keep in mind that on the vast majority of occasions, it is not possible to obtain correctly labeled datasets from production networks to train models. Therefore, the above results validate DOROTHEA as a suitable tool for collecting sampled flow datasets. This is a good starting point for future research aimed at improving security in networks with high traffic loads.

## Conclusions

Anomaly detection has shown promising results in detecting malicious traffic using complete network packets. Nevertheless, networks with a high traffic load can only carry out a partial packet analysis. Such networks often use a flow-based protocol such as NetFlow. However, even using NetFlow, the traffic load handled by some routers is so high that they have to sample packets to generate flow data.

In this work, the OC-SVM and iForest models have been trained and tested with NetFlow-based datasets with a sampling rate of 1 out of 1,000 packets to detect network anomalies.

The novelty detection technique was employed, so the training datasets only contained benign traffic. We used several datasets. On the one hand, synthetic flow datasets were gathered with DOROTHEA. On the other hand, flow datasets were gathered from RedCAYLE. The test datasets were balanced, containing benign and malicious flow data. Experiments showed that the OC-SVM model has high malicious traffic detection power, with an accuracy score above 91.5% and a FAR score below 1.4% in both networks.

Two conclusions can be drawn from the experiments carried out.

1. First, OC-SVN has a high novelty detection rate with a low false alarm rate. Consequently, it is possible to detect anomalies using novelty detection in NetFlow data with a sample rate of 1 out of 1,000 packets. Therefore, we can confirm that it is possible to see malicious traffic in production networks such as RedCAYLE and similar networks, improving their security.
2. On the other hand, the results obtained with the synthetic datasets collected with DOROTHEA are similar to those obtained with the datasets from RedCAYLE. Therefore, we affirm that DOROTHEA’s datasets are valid for training models for anomaly detection. Furthermore, generating datasets with DOROTHEA can be a starting point for other researchers to continue improving the security of networks with a high traffic load since it is often not possible to gather labeled flow datasets from such a realistic scenario.

In future work, we intend to experiment with deep learning-based models to try to improve the results obtained by the OC-SVN model and thus further improve the security of networks such as RedCAYLE.

## Data Availability

The datasets generated and analyzed during the current study are available in the RoboticsGroup repository: https://github.com/uleroboticsgroup/MoEv/tree/Anomalies.
